# Efficacy of pharmacological therapies in reducing outflow tract obstruction in patients with obstructive hypertrophic cardiomyopathy: a systematic review and meta-analysis

**DOI:** 10.1093/ehjcvp/pvaf036

**Published:** 2025-05-16

**Authors:** Kamal Awad, Milagros Pereyra Pietri, Juan M Farina, Girish Pathangey, Mohammed Tiseer Abbas, Isabel G Scalia, David Le Couteur, Susan Wilanksy, Steven J Lester, Steve R Ommen, Jeffrey B Geske, Reza Arsanjani, Chadi Ayoub

**Affiliations:** Department of Cardiovascular Medicine, Mayo Clinic, 5777 E Mayo Blvd, Phoenix, AZ 85054, USA; Department of Cardiovascular Medicine, Mayo Clinic, 5777 E Mayo Blvd, Phoenix, AZ 85054, USA; Department of Cardiovascular Medicine, Mayo Clinic, 5777 E Mayo Blvd, Phoenix, AZ 85054, USA; Department of Cardiovascular Medicine, Mayo Clinic, 5777 E Mayo Blvd, Phoenix, AZ 85054, USA; Department of Cardiovascular Medicine, Mayo Clinic, 5777 E Mayo Blvd, Phoenix, AZ 85054, USA; Department of Cardiovascular Medicine, Mayo Clinic, 5777 E Mayo Blvd, Phoenix, AZ 85054, USA; ANZAC Research Institute, Concord Hospital, Hospital Road, Concord, NSW 2139, Australia; Department of Cardiovascular Medicine, Mayo Clinic, 5777 E Mayo Blvd, Phoenix, AZ 85054, USA; Department of Cardiovascular Medicine, Mayo Clinic, 5777 E Mayo Blvd, Phoenix, AZ 85054, USA; Department of Cardiovascular Medicine, Mayo Clinic, 200 First St. SW, Rochester, MN 55905, USA; Department of Cardiovascular Medicine, Mayo Clinic, 200 First St. SW, Rochester, MN 55905, USA; Department of Cardiovascular Medicine, Mayo Clinic, 5777 E Mayo Blvd, Phoenix, AZ 85054, USA; Department of Cardiovascular Medicine, Mayo Clinic, 5777 E Mayo Blvd, Phoenix, AZ 85054, USA

**Keywords:** Hypertrophic cardiomyopathy, Left ventricular outflow tract obstruction, Medical treatment

## Abstract

**Aims:**

Significant advancements have been made in the management of obstructive hypertrophic cardiomyopathy (oHCM), yet the extent of left ventricular outflow tract (LVOT) gradient reduction achieved with commonly used pharmacological therapies [beta-blockers (BBs), calcium channel blockers (CCBs), disopyramide, and cardiac myosin inhibitors (CMIs)] relative to each other is still unclear.

**Methods and results:**

PubMed and Scopus were searched up to September 2024. Clinical trials or observational studies that assessed the changes associated with BBs, CCBs, disopyramide, or CMIs in LVOT gradient at rest or with provocation in patients with oHCM were included. Mean changes in LVOT gradients were pooled as mean differences (MD) with 95% confidence intervals (CIs) in a random-effects model. Thirty-seven studies, with 44 arms and 1898 patients, were included in the analysis. At the therapeutic class level, pooled analysis showed that disopyramide was associated with the highest reduction in LVOT gradient at rest [MD: −43.5 (95% CI, −51.6 to −35.3)], followed by CMIs [MD: −34.8 (95% CI, −40.6 to −29.0)], BBs [MD: −20.7 (95% CI, −29.4 to −12.0)], and then CCBs [MD: −14.7 (95% CI, −23.3 to −6.1)], inter-action *P* < 0.01. Within CMIs, mavacamten had a higher effect than aficamten on gradient reduction; among the included BBs, metoprolol showed the highest gradient reduction, while among CCBs, verapamil was the most effective (inter-action *P* < 0.01). Similar results were observed for provocable LVOT gradients.

**Conclusion:**

Pharmacological therapies effectively reduced LVOT gradients in oHCM patients to varying degrees, with disopyramide and CMIs showing the highest effect, followed by BBs and CCBs.

## Introduction

Hypertrophic cardiomyopathy (HCM) is the most common inherited heart disease and associated left ventricular outflow tract (LVOT) obstruction (LVOTO) can result in symptoms (e.g. dyspnoea, angina, or syncope) and reduced functional capacity.^1,2^ Approximately two thirds of patients with HCM have LVOTO either at rest or with provocation, termed obstructive hypertrophic cardiomyopathy (oHCM).^3^ Negative ionotropic therapy with beta-blockers (BBs), calcium channel blockers (CCBs), and disopyramide have traditionally been used as initial medical therapy to treat oHCM. These first line therapies may improve pre-load, have a negative chronotropic and inotropic effects, but may also cause side effects. In cases where maximally titrated doses of these agents fail to alleviate symptomatic LVOTO, septal reduction therapy (SRT) with myectomy or alcohol septal ablation had traditionally been undertaken. In the current era, the novel class of cardiac myosin inhibitors (CMI) have changed the landscape and are now used in HCM patients who have persistent LVOTO despite first-line therapy, offering an alternative to invasive SRT in many patients.

Despite widespread use of negative inotropic therapy and increasing utilization of CMIs, there is, to the best of our knowledge, no study that comprehensively assessed their comparative efficacy in reducing LVOT gradients in oHCM. Each negative inotropic medication has potential side effects, and CMIs also carry additional costs and a greater surveillance burden. In light of this, we conducted a systematic review and meta-analysis to compare current guideline-recommended pharmacological therapies (both by class and as separate agents) to better understand their real-world comparative efficacy and relative reduction in LVOT gradients in patients with oHCM.

## Methods

The study was conducted in adherence to the preferred reporting items for systematic reviews and meta-analyses (PRISMA) statement (see Supplementary material online, *Table S1*).^4^ An *a priori* study protocol was established and applied. No Institutional Review Board approval or patient informed consent was required for the systematic review of the literature and meta-analysis.

### Literature search strategy

PubMed/Medline and Scopus were interrogated up to September 2024 for a combination of relevant keywords and Medical Subject Headings (MeSH) terms. The full search strategy is presented in Supplementary material online, *Table S2*. The search was limited to English and human studies. Multiple publications of retrieved articles (i.e. with the same population) were acquired in order to include the most complete or up-to-date study results. Moreover, a manual search of the reference lists of the included studies was conducted to ensure that no relevant studies were overlooked. The first selection was made by reading titles/abstracts and the final selection by reading the full articles. Articles were screened by two independent reviewers (K.A. and M.P.P.), and any discordance was resolved by the opinion of a third reviewer (C.A. or J.M.F.). Duplicates were identified and removed using Endnote 21 (Clarivate, Philadelphia, PA, USA).

### Study selection criteria

Original studies were included if met the following criteria: Clinical trials (including randomized or non-randomized approach), experimental studies (before/after treatment), or observational studies that assessed LVOT maximal instantaneous gradient changes (at rest or with provocation) associated with BBs, CCBs, disopyramide or CMIs (specifically mavacamten or aficamten) in patients with oHCM. Studies that reported a provokable LVOT gradient either post-exercise or with Valsalva maneuver were included, with either value used in the analysis as a provokable LVOT gradient. If a study reported gradients from both provocation methods, the measurement with the higher baseline gradient or assessed in a larger sample was chosen for the analysis. If both methods were assessed in the same number of patients, the one with the higher baseline measurement was included. There is data showing close correlation with invasively measured and echocardiographic peak LVOT gradients in HCM, providing validity for the comparison of both hemodynamic and echocardiographic measures in this meta-analysis.^5^

Exclusion criteria included one or more of the following: non-English articles, absence of a full peer reviewed published manuscript, reviews, book chapters, case reports, retracted articles, studies conducted *in vitro* or in animals, studies with insufficient data for analysis of LVOT gradients, studies that included infants or children, and articles that included the same population of a previous study or trial. Studies that included patients that underwent invasive SRT (e.g. alcohol septal ablation or myectomy) were excluded to better assess the actual efficacy of the respective pharmacological treatments. Studies that included practolol or dihydropyridine CCBs (e.g. nifedipine) were also excluded given the former was withdrawn from the market due to reported adverse effects and the latter due to vasodilatory effects is not used clinically for LVOT gradient reduction in oHCM.^6,7^

### Data extraction

Data were extracted independently by 2 reviewers (K.A. and M.P.P.) from publications that met the inclusion criteria from the second read using a standardized data extraction electronic form. Discrepancies were resolved by consensus between the two reviewers and the senior investigator. Data that were abstracted included study and patient-related characteristics as follows: first author’s name, year of publication, study location, study design, sample size, treatment type, dose and duration, age, sex, background/concurrent treatment, baseline and follow-up LVOT gradients, and adverse events (AEs).

### Risk of bias assessment

The risk of bias in non-randomized studies of interventions (ROBINS-I) tool was used for the risk of bias assessment of the included non-randomized and observational studies.^8^ This tool includes seven domains and rates the overall risk of bias as low, moderate, serious, critical, or unclear. Moreover, the Cochrane risk-of-bias tool (RoB2) was used for risk of bias assessment of the included RCTs.^9^ This tool assesses the following items: randomization process, effect of assignment to intervention, effect of adhering to intervention, missing outcome data, measurement of the outcome, and selection of the reported result. Judgment is either low some concerns or high risk of bias. Two reviewers (M.P.P. and G.P.) independently performed this assessment, and disagreements were resolved by discussion between the two reviewers and the senior investigator.

### Quantitative data synthesis

The change in LVOT gradient values (across treatment duration from baseline or across the pharmacological intervention group compared with placebo, whenever reported) were collected in mmHg. If not reported, the mean change in LVOT gradient after treatment were calculated as follows: (LVOT gradient value at end of follow-up)—(measurement at baseline). Their related standard deviations (SD) were also computed as follows: SD = square root [(SD_pre-treatment_)^2^ + (SD_post-treatment_)^2^−(2*R* * SD_pre-treatment_ * SD_post-treatment_)], assuming a correlation coefficient (*R*) of 0.5, as described before.^10,11^ The method by Wan *et al*.^12^ was used to calculate the mean and SD when the data were reported as median and range. Likewise, the method by Altman and Bland was used if values were reported as mean and standard error or confidence interval (CI).^11,13^

Mean changes in LVOT gradients (either after the treatment compared with the baseline agent or the relative changes in the treatment group compared with placebo, whenever reported) were pooled as mean differences (MD) with 95% CI in a random-effects model, given the assumed heterogeneity among the included studies due to variations in study design and patient characteristics. The pooled analysis was performed based on sub-grouping according to treatments classes (i.e. BBs, CCBs, disopyramide, or CMIs) and the individual drug agents, to address heterogeneity and to quantify the efficacy of each treatment separately. A *P* value for inter-action below 0.1 implies a statistically significant sub-group effect.^14^ A sensitivity analysis excluding the acute effects studies (i.e. those that assessed the immediate effects of intravenous treatments) was also conducted to evaluate the long-term, sustained therapeutic effects. However, this sensitivity analysis was applied only to the treatment classes, not to the individual drug agents, due to the low number of studies that assessed each individual drug agent after excluding the acute effects studies.

Inter-study heterogeneity was assessed by visual inspection of the forest plots and quantified by I^2^ and χ^2^ tests. Heterogeneity was interpreted according to the Cochrane Handbook recommendations. The I^2^ test was read as follows: 0% to 40%, low heterogeneity; 30% to 60% may represent moderate heterogeneity; and 50% to 90%, may indicate substantial heterogeneity. A χ^2^ test *P* value below 0.1 was considered significant heterogeneity. Potential publication bias was evaluated by visual inspection of Begg’s funnel plot asymmetry and statistically confirmed by Egger’s regression test.^15^ Analyses were performed using R software (v 4.3.2, R Foundation for Statistical Computing, Vienna, Austria).

## Results

### Flow and characteristics of included studies

**Figure 1:**
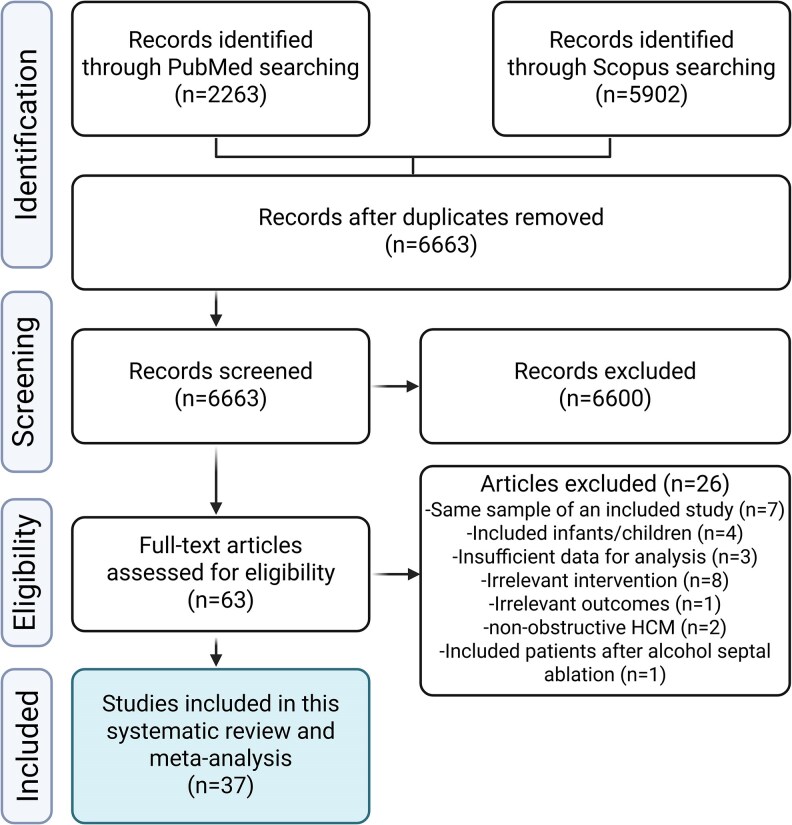
Study flow diagram showing the selection process of studies included in the systematic review and meta-analysis.

A total of 8165 records were identified through the literature search. After removal of duplicates and the two-step screening approach, 37 articles [with 44 arms, including 11 clinical trials (7 randomized and 4 non-randomized), 13 experimental studies (before/after treatment), and 13 observational studies (7 prospective and 6 retrospective cohorts), Supplementary material online, *Table S3*] met our inclusion criteria and were included in the analysis. *Figure 1* shows the PRISMA flow diagram for the current study along with the reasons for excluded studies. The current analysis included 1898 patients with oHCM. The average age of the included sample was 51 years (9.7) and 57% of subjects were male. The median follow-up was 13.6 months (IQR 7.4). The publication year of the included studies ranged from 1979 up to 2024. *Table 1* shows the baseline characteristics of the included studies. In addition, Supplementary material online, *Table S4* summarize the reported AEs of the included drugs.

**Table 1:** Characteristics and baseline parameters of the studies included in the meta-analysis

Study	Study design	Sample	Intervention	Dose	Concurrent treatments	Follow-up time	Age**	Males**	Baseline rest LVOT gradient	Baseline provokable LVOT gradient
Monda *et al.*^16^	Observational retrospective cohort study	92	Bisoprolol	5.7 (3.2) mg	None	6 months (3 to 7)	49.9 (16.5)	54 (59%)	69 (15)	NA
Dybro *et al.*^17^	RCT	29	Metoprolol	50, 100, 150 mg	None	6 weeks	60 (11)	18 (62%)	62.3 (46)	113.66 (90.44)
Haruki *et al.*^18^	Observational retrospective cohort study	33	Disopyramide	271 (81) mg/day	27 BBs and 9 CCBs	9.58 (7.14) years	56.8 (16.1)	10 (30.3%)	74.5 (26.4)	NA
Sherrid *et al.*^19^	Observational prospective cohort study	141	Disopyramide	600 mg/day	BBs or verapamil	4.5 (3.6) years	56.7 (15)	153 (51%)	59 (46)	NA
Nistri *et al.*^20^	Observational prospective cohort study	27	Nadolol	80 mg/day	None	12 (4) months	36 (15)	22 (81%)	NA	87 (29)
Kajimoto *et al.*^21^	Non-randomized trial	24	Disopyramide	1.0 mg/kg/5min	None	5 min post-administration	55 (42–58)*	NA	92.3 (32.9)	NA
Kajimoto *et al.*^21^		12	Propranolol	0.20 mg/kg/5min	None	5 min post-administration	55 (42–58)*	NA	93.9 (23.1)	NA
Kajimoto *et al.*^21^		12	Verapamil	0.10 mg/kg/5min	None	5 min post-administration	55 (42–58)*	NA	86.9 (23.3)	NA
Sherrid *et al.*^22^	Observational retrospective cohort study	78	Disopyramide	600 mg/day	70 BBs, 35 CCBs, and 11 both	3.1 (2.6) years	47 (20)	60 (51%)	75 (33)	NA
Sherrid *et al.*^22^		40	Disopyramide	600 mg/day					73 (35)	NA
Betocchi *et al.*^23^	Experimental study (before/after treatment)	16	Diltiazem	0.25 mg/kg over 2 min, then 0.014/mg/kg/min over 10 min	None (withdrawn before the study)	After 10 min of the infusion	38	11 (68.7%)	46 (36)	NA
Matsubara *et al.*^24^	Experimental study (before/after treatment)	6	Disopyramide	50 mg or 100 mg	None (withdrawn before the study)	After administration of the drug	55 (8)	3 (50.0%)	100 (45)	NA
Kimball *et al.*^25^	Experimental study (before/after treatment)	25	Disopyramide	100 mg	None (withdrawn before the study)	After administration of the drug	40 (18–70)*	12 (48%)	86 (34)	124 (33)
Dimitrow and Dubiel^26^	Experimental study (before/after treatment)	20	Pindolol	5 mg/twice daily	Verapamil	6 months	37 (8)	15 (75%)	29 (30)	NA
Millaire *et al.*^27^	Experimental study (before/after treatment)	9	Disopyramide	500 (50) mg/day	NA	1 month	39 (14)	6 (66.7%)	83 (51)	107 (37)
Pollick *et al.*^28^	Experimental study (before/after treatment)	43	Disopyramide	2 mg/kg	NA	After completion of drug infusion	44	25 (58.1%)	74 (34)	138 (44)
Pollick *et al.*^29^	RCT	10	Disopyramide	150 mg four times a day	NA	4 days	48	8 (80%)	61 (20)	NA
Pollick *et al*^28^		10	Propranolol	40 mg four times a day	NA	4 days	48	8 (80%)	61 (20)	NA
Sherrid *et al.*^30^	Experimental study (before/after treatment)	7	Disopyramide	150 mg every 6 h	None	23 days	64	3 (42.8%)	63.57 (14.66)	NA
Anderson *et al.*^31^	Experimental study (before/after treatment)	15	Verapamil	690 mg/day	All on propranolol	6 months	52	11 (73.3%)	57 (155)	114 (232.37)
Bonow *et al.*^32^	Experimental study (before/after treatment)	10	Verapamil	0.007, 0.014, and 0.021 mg/kg/min for 10 min each	None	At the completion of drug infusion	47	7 (50.0%)	62 (27)	NA
Tendera *et al.*^33^	Experimental study (before/after treatment)	8	Verapamil	0.1 mg/kg over two minutes, followed by infusion of 0.01 mg/kg/min.	None	After 10 min of the infusion	33 (10)	13 (81.2%)	50 (37)	NA
Landmark *et al.*^34^	Non-randomized trial	6	Propranolol	5 mg intravenously over 5 min	None	After 10 min of the injection	45 (26–67)	NA	81 (22)	NA
Pollick *et al.*^35^	Experimental study (before/after treatment)	4	Disopyramide	100 mg (IV), 300 mg (acute/24 h), 200 mg 4 times day (maintenance)	None	After the injection	23–62	3 (60.0%)	50.25 (24.68)	120.5 (54.24)
Storstein *et al.*^36^	non-randomized trial	7	Pindolol	10 mg	None	After the injection	39.1	4 (57.1%)	35.4 (35.8)	NA
Kaltenbach *et al.*^37^	Experimental study (before/after treatment)	9	Verapamil	480 mg/day	None	15 months (range 4–24 months)	19–44	18 (81.8%)	21.55 (27.35)	51.11 (19.83)
Rosing *et al.*^38^	Experimental study (before/after treatment)	14	Verapamil	0.007, 0.014 and 0.021 mg/kg/min.	Propranolol	After 10 min of the infusion	44 (3)	16 (59.2%)	86 (26.2)	90 (27.71)
Todde *et al*.^39^	Observational prospective cohort study	47	Disopyramide	220 (29) mg/day	41 BBs and 6 CCBs	4.4 (3.6) years	52 (14)	36 (57%)	78 (25)	NA
Todde *et al*.^39^		15	Disopyramide	223 (46) mg/day	11 BBs and 4 CCBs		50 (13)	36 (57%)	79 (38)	NA
Abood *et al*.^40^	Observational retrospective cohort study	31	Mavacamten	5 mg	31 BBs, 25 CCBs, and 6 Disopyramide	12 weeks	58 (16.5)	16 (51.6%)	NA	77.7
Desai *et al*.^41^	Observational prospective cohort study	150	Mavacamten	5 mg	124 BBs, 36 CCBs, and 7 Disopyramide	12 weeks	65 (12)	71 (47.0%)	41 (33)	72 (43)
Ramonfaur *et al.*^42^	Observational prospective cohort study	66	Mavacamten	2.5, 5, 10, and 15 mg/day	59 BBs, 19 CCBs, and 3 Disopyramide	18 months	58.8 (15)	31 (47%)	56 (43)	107 (44)
Reza *et al*.^43^	Observational retrospective cohort study	80	Mavacamten	5 mg	31 BBs, 8 CCBs, 3 Disopyramide	36 weeks	62.9 (14.3)	45 (46%)	56.6 (39.1)	78.4 (33.0)
Roehl *et al*.^44^	Observational retrospective cohort study	34	Mavacamten	<2.5 or >2.5 mg	NA	12 weeks	NA	NA	72.8(20.2), 0.007 (long-term)	NA
Wessly *et al*.^45^	Observational prospective cohort study	15	Mavacamten	5 mg	NA	30 days	70 (13)	6 (40.0%)	45 (23)	79 (28)
Tian *et al.*^46^	RCT	81	Mavacamten	2.5 mg	BBs (72 in control, 48 in mavacamten), CCBs (6 in control, 4 in mavacamten), others (3 in control, 2 in mavacamten)	30 weeks	51.9 (11.9)	58 (71.6%)	74.6 (35.1)	106.8 (43.2)
Olivotto *et al.*^47^	RCT	251	Mavacamten	5 mg	BBs (189 in control, 94 in mavacamten), CCBs (42 in control, 25 mavacamten)	30 weeks	58.5 (12.2)	149 (59%)	52 (59)	86 (34)
Heitner *et al.*^48^	Non-randomized trial	11	Mavacamten	10–20 mg/day	No background therapy	12 weeks	56 (22–70)*	7 (64%)	60 (28)	103 (50)
Heitner *et al.*^48^		10	Mavacamten	2–5 mg/day	9 BBs	12 weeks	58 (26–67)*	5 (50%)	86 (63)	86 (43)
Desai *et al.*^49^	RCT	112	Mavacamten	2.5, 5, 10, or 15 mm/day	106 (BBs, CCBs, Disopyramide or combination)	16 weeks	59.8 (14.2)	29 (51.8%)	51.2 (31.4)	82.5 (34.7)
Maron *et al*.^50^	RCT	282	Aficamten	20 mg	241 (BBs, CCBs, Disopyramide)	24 weeks	59.2 (12.6)	167 (59%)	54.8 (27)	82.9 (32)
Maron *et al.*^51^	RCT	27	Aficamten	5–30 mg	All on either BBs or CCBs	12 weeks	57 (26–33)	13 (46.4%)	53.8 (cohort 1), 58.2 (cohort 2)	74.4 (cohort 1), 82.3 (cohort 2)
Owens *et al.*^52^	Observational prospective cohort study	13	Aficamten	15 mg	10 BBs, 2 CCBs, and 1 both	12 weeks	62 (58–65)	6 (46.1%)	45(38–62)	86 (78–103)

Continuous variables are reported as mean (standard deviation) or *median (range or inter-quartile range), and categorical variables are reported frequency (percentage). **If the data on the included sub-group for these variables is not reported, we used the data for the whole study sample. RCT, randomized controlled trial; BB, beta-blockers; CCB, calcium channels blockers; LVOT, left ventricular outflow tract; NA, not available.

### Risk of bias in the included studies

According to the ROBINS-I tool, most of the included observational/non-randomized studies (22 out of 30) showed a high risk of bias mainly due to the incomplete control for the potential confounders; these studies were predominately older studies from the early oHCM era without control groups. Meanwhile, most of the included clinical trials showed a low risk of bias according to the RoB 2 tool. Details of the risk of bias assessment using both tools are shown in Supplementary material online, *Table S5*.

### Analyses results on the effect of included treatments on LVOT gradients

**Figure 2:**
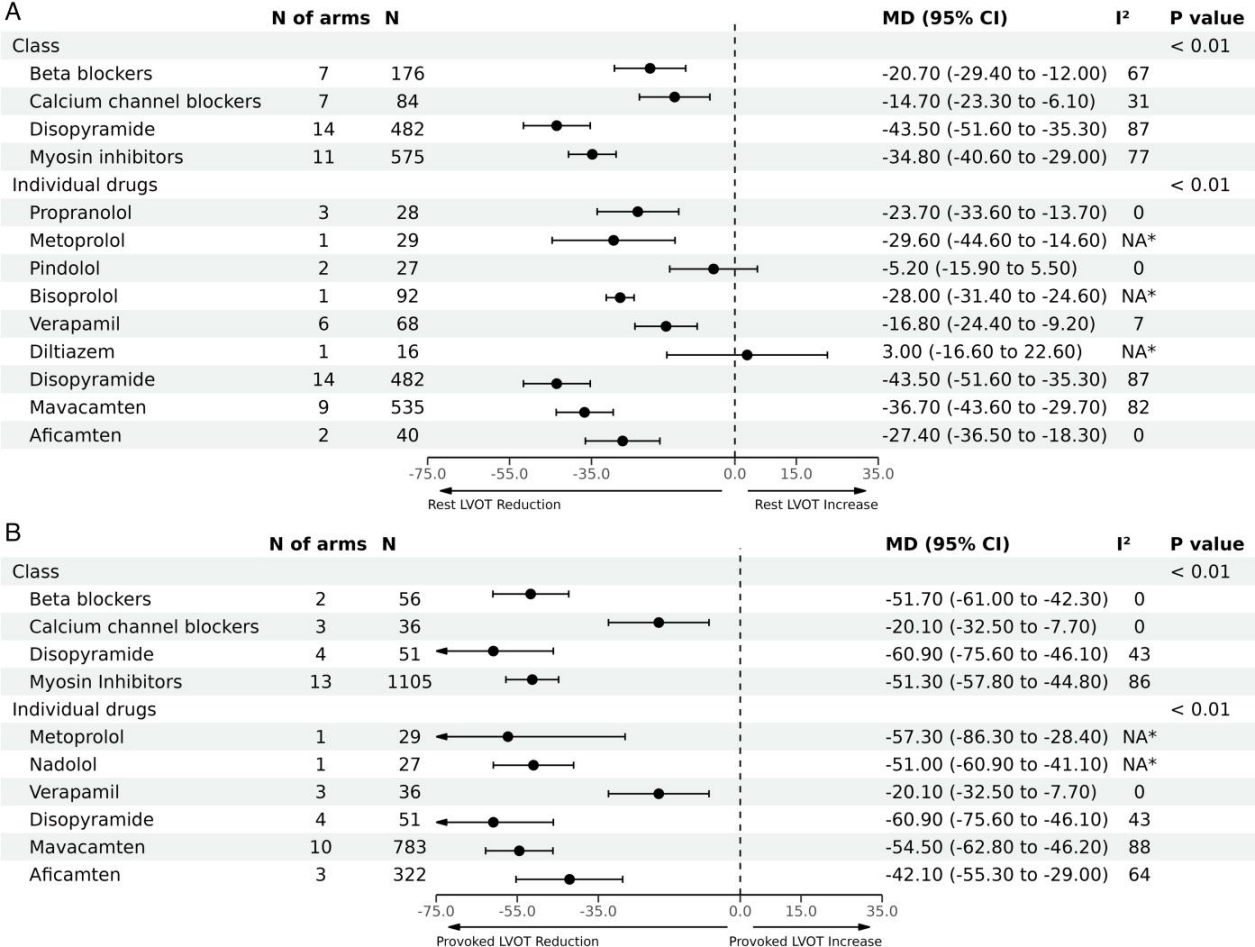
Pooled analyses by therapeutic class and individual agent for mean difference in left ventricular outflow tract (LVOT) gradient reduction. Panel (*A*) demonstrates mean difference in reduction of resting LVOT gradients, and Panel (*B*) demonstrates mean difference in reduction of LVOT gradients with provocation. *Not applicable because only one study was included in this analysis.

At the therapeutic class level, pooled analyses showed that disopyramide and CMIs were associated with the highest reduction in LVOT gradient at rest [MD: −43.5 mmHg (95% CI, −51.6 to −35.3), *n* = 482] and [MD: −34.8 mmHg (95% CI, −40.6 to −29.0), *n* = 575], respectively; followed by BBs [MD: −20.7 mmHg (95% CI, −29.4 to −12.0), *n* = 176], then CCBs [MD: −14.7 mmHg (95% CI, −23.3 to −6.1), *n* = 84], inter-action *P* < 0.01 (*Figure 2*). A high degree of heterogeneity was observed across the pooled estimates for the included classes (I^2^ ranged from 67% up to 87%, *Figure 2*), except for the CCBs (I^2^ = 31%).

On an individual agent level within each class, mavacamten had higher effect on LVOT gradients [MD: −36.7 mmHg (95% CI, −43.6 to −29.7), *n* = 535] than aficamten, although the latter is still under investigation and has smaller numbers available for pooling at this time. Metoprolol showed the highest reduction in LVOT gradient at rest among BBs [MD: −29.5 mmHg (95% CI, −44.6 to −14.6), *n* = 29], whilst among CCBs, verapamil was the most effective [MD: −16.8 mmHg (95% CI, −24.4 to −9.2), *n* = 68], with very small number of patients available for diltiazem. The comparison among the individual agents was significant with inter-action *P* < 0.01, *Figure 2*. Significant substantial heterogeneity was observed for disopyramide (87%), and mavacamten (82%), while was insignificant for the other agents. Similar results were observed in terms of LVOT reduction with provocation (*Figure 2*). However, significant heterogeneity was only observed for CMIs (86%) at the class level and with mavacamten and aficamten at the level of individual agents (88% and 64%, respectively). Summary of the changes in LVOT gradients in each of the included studies is shown in *Table 2*.

**Table 2:** Summary of changes in left ventricular outflow tract gradient at rest and with provocation induced by the included interventions

Study	Intervention	Sample	LVOT gradient at rest					LVOT gradient with provocation				
			Change [MD (SE)]	≥50 mmHg pre-treatment	<50 mmHg post-treatment	≥30 mmHg pre-treatment	<30 mmHg post-treatment	Change [MD (SE)]	≥50 mmHg pre-treatment	<50 mmHg post-treatment	≥30 mmHg pre-treatment	<30 mmHg post-treatment
Monda *et al*.^16^	Bisoprolol	92	−28 (1.7)	NA	57 (62)	NA	33 (35.9)	NA	92 (100)	NA	92 (100)	NA
Dybro *et al*.^19^	Metoprolol	29	−29.6 (7.7)	NA	NA	NA	NA	−57.3 (14.8)	NA	NA	NA	NA
Haruki *et al*.^18^	Disopyramide	33	−43.1 (4.5)	NA	NA	33 (100)	NA	NA	NA	NA	NA	NA
Sherrid *et al*.^19^	Disopyramide	141	−41 (3.4)	NA	NA	NA	NA	NA	NA	NA	NA	NA
Nistri *et al*.^20^	Nadolol	27	NA	0 (0)	NA	0 (0)	NA	−51 (5)	25 (92.6)	19 (70.4)	27 (100)	14 (51.9)
Kajimoto *et al*.^21^	Disopyramide	24	−51.7 (6.2)	21 (87.5)	18 (75)	24 (100)	10 (41.7)	NA	NA	NA	NA	NA
Kajimoto *et al*.^21^	Propranolol	12	−17.1 (7.8)	12 (100)	3 (25)	12 (100)	0 (0)	NA	NA	NA	NA	NA
Kajimoto *et al*.^21^	Verapamil	12	−6.5 (6.7)	11 (91.7)	1 (8.3)	12 (100)	0 (0)	NA	NA	NA	NA	NA
Sherrid *et al*.^22^	Disopyramide	78	−35 (3.7)	78 (100)	NA	78 (100)	NA	NA	NA	NA	NA	NA
Sherrid *et al*.^22^	Disopyramide	40	−10 (5.2)	40 (100)	11 (27.5)	40 (100)	NA	NA	NA	NA	NA	NA
Betocchi *et al*.^23^	Diltiazem	16	3 (10)	NA	NA	11 (68.8)	NA	NA	NA	NA	NA	NA
Matsubara *et al*.^24^	Disopyramide	6	−74 (16.5)	5 (83.3)	5 (83.3)	6 (100)	5 (83.3)	NA	NA	NA	NA	NA
Kimball *et al*.^25^	Disopyramide	25	−59 (5.9)	NA	NA	NA	NA	−60 (6.6)	NA	NA	NA	NA
Dimitrow *et al*.^26^	Pindolol	20	−7 (6)	NA	NA	NA	NA	NA	NA	NA	NA	NA
Millaire *et al*.^27^	Disopyramide	9	−45 (14.9)	6 (66.7)	6 (66.7)	7 (77.8)	5 (55.6)	−38 (13.6)	7 (77.8)	4 (44.4)	7 (77.8)	2 (22.2)
Pollick *et al.*^28^	Disopyramide	43	−62 (4.5)	NA	41 (95.3)	40 (93)	37 (86)	−76 (9.9)	NA	NA	NA	NA
Pollick *et al*.^28^	Disopyramide	10	−56 (5.7)	NA	NA	NA	NA	NA	NA	NA	NA	NA
Pollick *et al*.^28^	Propranolol	10	−31 (8.4)	NA	NA	NA	NA	NA	NA	NA	NA	NA
Sherrid *et al*.^30^	Disopyramide	7	−50 (5.7)	6 (85.7)	7 (100)	7 (100)	6 (85.7)	NA	NA	NA	NA	NA
Anderson *et al*.^31^	Verapamil	15	−23 (43.8)	NA	NA	NA	NA	−29 (68.1)	NA	NA	NA	NA
Bonow *et al*.^32^	Verapamil	10	−30 (10)	7 (70)	7 (70)	9 (90)	5 (50)	NA	NA	NA	NA	NA
Tendera *et al*.^33^	Verapamil	8	−25 (11.8)	2 (25)	7 (87.5)	6 (75)	5 (62.5)	NA	NA	NA	NA	NA
Landmark *et al*.^34^	Propranolol	6	−24 (11)	6 (100)	NA	6 (100)	NA	NA	NA	NA	NA	NA
Pollick *et al*.^35^	Disopyramide	4	−48.75 (12)	2 (50)	4 (100)	3 (75)	4 (100)	−66.75 (23.4)	4 (100)	2 (50)	4 (100)	1 (25)
Storstein *et al*.^36^	Pindolol	7	4.6 (13.9)	NA	NA	NA	NA	NA	NA	NA	NA	NA
Kaltenbach *et al*.^37^	Verapamil	9	−12. 6 (7.9)	2 (22.2)	9 (100)	3 (33.3)	8 (88.9)	−25 (9.5)	5 (55.6)	7 (77.8)	8 (89)	6 (66.7)
Rosing *et al*.^38^	Verapamil	14	−22 (7.5)	NA	NA	14 (100)	3 (21.4)	−16 (8.5)	NA	NA	12 (85.7)	NA
Todde *et al*.^39^	Disopyramide	47	−35 (4.3)	NA	35 (74.5)	NA	NA	NA	NA	NA	NA	NA
Todde *et al*.^39^	Disopyramide	15	−13 (8.8)	NA	4 (26.7)	NA	NA	NA	NA	NA	NA	NA
Abood *et al*.^40^	Mavacamten	31	NA	NA	NA	NA	NA	−60.9 (4.5)	31 (100)	26 (83.9)	31 (100)	26 (83.9)
Desai *et al*.^41^	Mavacamten	150	−26 (0.2)	NA	NA	150 (100)	NA	−43 (0.3)	142 (94.7)	114 (76)	150 (100)	106 (70.7)
Ramonfaur *et al*.^42^	Mavacamten	66	−37 (4.9)	61 (92.4)	NA	61 (92.4)	NA	−80 (7.1)	61 (92.4)	NA	61 (92.4)	NA
Reza *et al*.^43^	Mavacamten	56	−38.6 (4.5)	NA	NA	56 (100)	48 (85.7)	−52.8 (3.8)	NA	NA	56 (100)	48 (85.7)
Roehl *et al.*^44^	Mavacamten	34	−26.2 (5.2)	NA	NA	NA	NA	NA	NA	NA	NA	NA
Wessly *et al.*^45^	Mavacamten	15	−40 (5.2)	NA	NA	NA	NA	−65 (6.4)	NA	NA	NA	NA
Tian *et al.*^46^	Mavacamten	81	−55 (7.2)	NA	NA	NA	NA	−70.29 (9.9)	81 (100)	32 (59) mavacamten vs. 3 (11) placebo	81 (100)	26 (48) mavacamten vs. 1 (3.7) placebo
Olivotto *et al.*^47^	Mavacamten	251	NA	NA	NA	NA	NA	−35.6 (3.9)	251 (100)	97 (79) mavacamten vs. 44 (34) placebo	251 (100)	74 (60) mavacamten vs. 22 (17.2) placebo
Heitner *et al.*^48^	Mavacamten	11	−47.8 (12.4)	NA	10 (90.9)	11 (100)	10 (90.9)	−89.5 (24.9)	11 (100)	10 (91)	11 (100)	8 (72.7)
Heitner *et al.*^48^	Mavacamten	10	−48.5 (17.5)	NA	NA	10 (100)	NA	−47.1 (17.9)	10 (100)	4 (40)	10 (100)	0 (0)
Desai *et al.*^49^	Mavacamten	112	−33.4 (4.5)	NA	NA	NA	NA	−37.2 (5.6)	NA	NA	NA	NA
Maron *et al.*^50^	Aficamten	282	NA	NA	NA	NA	NA	−50 (3.3	282 (100)	NA	282 (100)	70 (49) aficamten vs. 5 (3.6) placebo
Maron *et al*.^51^	Aficamten	27	−28 (7.2)	NA	NA	NA	NA	−43 (9.1)	NA	NA	NA	NA
Owens *et al.*^52^	Aficamten	13	−27 (6.1)	NA	NA	13 (100)	7 (53.8)	−28 (8.9)	NA	NA	NA	NA

Categorical variables are reported frequency (percentage).

MD, mean difference; SE, standard error; LVOT, left ventricular outflow tract; NA, not available.

### Results of the sensitivity analysis excluding the acute effects studies

**Figure 3:**
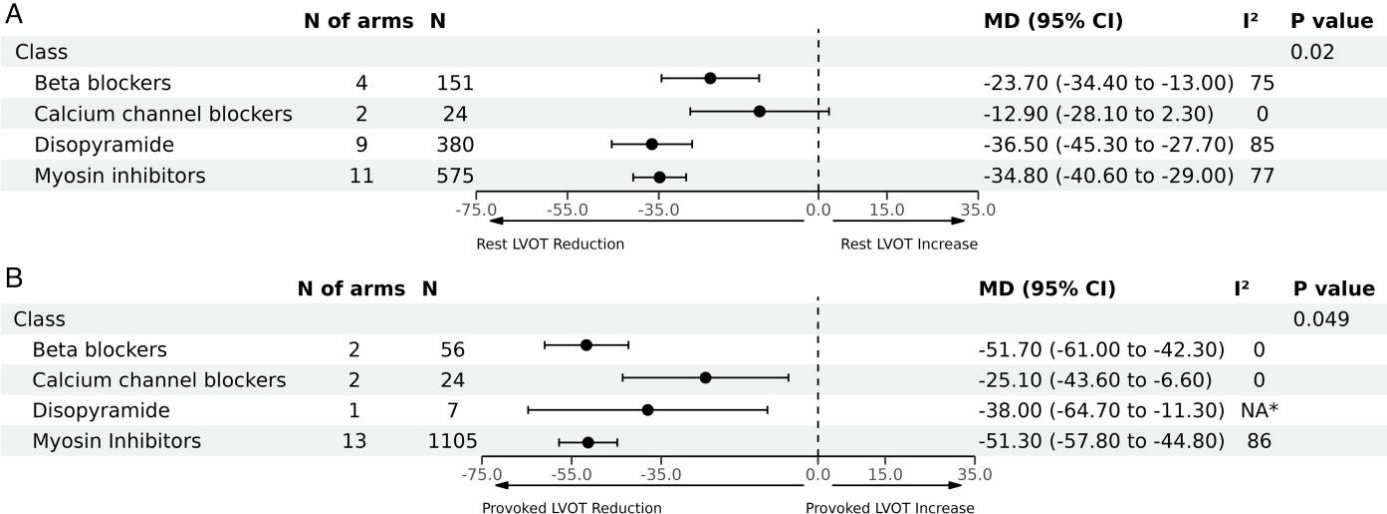
Pooled analyses by therapeutic class for the mean difference in left ventricular outflow tract (LVOT) gradient reduction after a sensitivity analysis excluding studies that measured the immediate effects of the relevant treatments. Panel (*A*) demonstrates mean difference in reduction of resting LVOT gradients, and Panel (*B*) demonstrates mean difference in reduction of LVOT gradients with provocation. *Not applicable because only one study was included in this analysis.

As a sensitivity analysis, we excluded studies with measurement of immediate effects of the relevant treatments, which resulted in 26 articles (with 31 arms) remaining for analysis. Consistent with the previous results, disopyramide and CMIs were associated with the highest reduction in LVOT gradient at rest [MD: −36.5 mmHg (95% CI, −45.3 to −27.7), *n* = 380] and [MD: −34.8 mmHg (95% CI, −40.6 to −29.0), *n* = 575], respectively; followed by BBs [MD: −23.7 mmHg (95% CI, −34.4 to −13.0), *n* = 151]. However, the effect of CCBs on LVOT gradient at rest became insignificant [MD: −12.9 mmHg (95% CI, −28.1 to 2.3), *n* = 24], inter-action *P* < 0.01 (*Figure 3*). Significant heterogeneity was observed across the pooled estimates for the included classes (I^2^ ranged from 75% up to 85%, *Figure 3*), except for the CCBs (I^2^ = 0).

In a similar sensitivity analysis for LVOT gradients with provocation, CMIs and BBs were associated with the highest reduction [MD: −51.3 mmHg (95% CI, −57.8 to −44.8), *n* = 1105] and [MD: −51.7 mmHg (95% CI, −61.0 to −42.3), *n* = 56], respectively; followed by disopyramide [MD: −38.0 mmHg (95% CI, −64.7 to −11.3), *n* = 7], then CCBs [MD: −25.1 mmHg (95% CI, −43.6 to −6.6), *n* = 24], inter-action *P* = 0.049 (*Figure 3*). Significant heterogeneity was observed only in terms of CMIs (I^2^ = 86%).

### Publication bias

The funnel plots for LVOT gradient at rest and during provocation appeared symmetrical, suggesting no significant publication bias (see Supplementary material online, *Figure S1*). This observation was further supported by the Egger’s test, which yielded *P*-values of 0.74 for the resting gradient and 0.5 for the provoked gradient, indicating no evidence of publication bias.

## Discussion

In this systematic review and meta-analysis comparing the effect of currently available pharmacological therapies on LVOT gradient reduction in patients with oHCM, disopyramide and CMIs were associated with the highest mean reduction in LVOT gradient followed by BBs, and CCBs, both on resting and provokable gradients. These results were maintained in a sensitivity analysis assessing only the oral treatment regimens on the resting LVOT gradient, except for CCBs where the effect became insignificant in the setting of the limited number of studies that assessed CCBs in the sensitivity analysis (i.e. only 2 studies). Whereas for LVOT gradients with provocation, CMIs and BBs were associated with the highest reduction, followed by disopyramide then CCBs.

Although prior meta-analyses have assessed the effect of CMIs on LVOT gradients^53^ and compared the overall effect of structured physical exercise programmes with pharmacologic therapy in general and invasive therapies on LVOT gradient reduction,^54^ to our knowledge this is the first systematic review and meta-analysis to assess and report the degree of reduction of LVOTO among all available pharmacologic classes and agents with inter-action testing for oHCM management including negative inotropic agents and CMIs.

Guideline directed clinical practice utilizes negative ionotropic therapy for treatment of symptomatic oHCM in the first instance, and if LVOTO persists despite maximal dose up-titration, then either a second negative inotropic agent or a CMI are added. Specifically, American Heart Association/American College of Cardiology (AHA/ACC) and European Society of Cardiology (ESC) guidelines recommend BBs as a first line to relieve symptoms caused by LVOTO.^2,55,56^ Non-dihydropyridine CCBs (either verapamil or diltiazem) are recommended as alternatives if BBs are ineffective despite using maximum tolerated doses, or if not tolerated because of side effects.^57^ If symptomatic LVOTO persists despite first line therapy, the AHA/ACC 2024 guideline recommend that treatment with disopyramide, CMI, and SRT (either percutaneous alcohol septal ablation or surgical myectomy) should all be discussed with the patient.^2^ The advent of CMIs has been suggested to reduce eligibility or need for SRT.^58^ The ESC guidelines follow a similar approach, but they reserve SRT for patients who continue to experience symptoms despite receiving maximal medical therapy.^56^ In addition, surgical myectomy may need to be considered in patients who have other indications for surgery, such as primary mitral valve disease.

Choosing between these treatment options should involve shared decision-making with the patient and is often influenced by clinician preference and experience.^55^ Although these classes and several agents therein are used pharmacologically to treat LVOTO, there has been no study to date comparing their relative efficacy on the degree of LVOT gradient reduction. Such data are of clinical importance, as it may guide clinician decision-making regarding therapy. The findings from this meta-analysis suggest that disopyramide and CMIs are associated with a greater reduction in LVOT gradient compared with BBs and CCBs. BBs appeared to have a greater effect on LVOT gradient reduction than CCBs within the negative inotrope group of medications, and among BBs, metoprolol was associated with the greatest LVOT gradient reduction. Among CCBs, there was data to support the use of verapamil (albeit with lower comparative effect on LVOT gradients compared with other agents); interestingly diltiazem, a commonly used CCB in oHCM, did not show significant reduction in LVOT gradient at rest. However, this finding is restricted by limited data with only one study evaluating the effects of this specific agent and should be considered with caution.

Disopyramide appeared to have a slightly greater effect on LVOT gradient reduction than CMIs, and this data are important as disopyramide represents a cost-effective alternative to CMIs for patients who do not achieve a satisfactory response with BBs and CCBs.^59^ CMIs in addition to greater cost also have an associated significant burden of echocardiographic surveillance for ejection fraction during treatment. However, precautions are required with the use of disopyramide, as it prolongs the QT interval, thereby increasing the risk of arrhythmias^55^; in addition, disopyramide may have anti-cholinergic gastrointestinal side effects.^60^

Among CMIs, this study also found that mavacamten may result in a greater mean reduction in LVOT gradients compared with aficamten. However, this finding is limited by the smaller number of studies currently available for aficamten. Aficamten has been suggested to have a superior pharmacologic profile given its shorter half-life and fewer drug inter-actions compared with mavacamten. This in turn may make aficamten an acceptable alternative option once further data accrues for its efficacy.^61^ CMIs, particularly mavacamten, in addition to LVOT gradient reduction and symptoms relief, may additionally improve cardiac remodelling, with potential disease modifying effect by specifically targeting the underlying pathophysiologic mechanisms in oHCM^62^; such a benefit over and above degree of LVOT gradient reduction may not be present in the negative inotropic therapy group, and is beyond the scope of this meta-analysis to assess.

A higher efficacy of metoprolol was noted in our study; however, prior data on direct head-to-head comparisons between this agent and other BBs for LVOT reduction is scarce. The greater effectiveness of metoprolol in reducing LVOT gradients is likely related to both its pharmacokinetic and pharmacodynamic properties, including its cardioselectivity and predominant action as a selective beta-1 receptor blocker. As such, it may exert stronger negative chronotropic and inotropic effects, thereby enabling a longer diastolic filling period and a greater reduction in LVOT gradients.^63,64^ Similarly, the potential higher efficacy of verapamil in reducing LVOT gradients in oHCM compared with the other non-dihydropyridine CCB can be explained by pre-clinical studies demonstrating that verapamil exerts a stronger negative inotropic and chronotropic effect.^65^ Verapamil may also increase myocardial oxygen delivery and decrease sympathetic nervous system activity to a greater extent, which could further enhance its ability to reduce the LVOT gradient compared with the other CCB.^66^

Findings herein have relevant clinical implications. This comprehensive and relative comparison of all available agents for oHCM in this meta-analysis may help guide physician decision-making regarding specific agent and classes of drugs for initial therapy for oHCM. It may also inform planning of future trials for oHCM therapies for comparison arms. Specifically, within BBs, which are first line agents, metoprolol appears to have the greatest effect on LVOTO gradients, and pindolol, which is occasionally used has weaker evidence for its efficacy. CCBs appear to be less efficacious than BBs, and the findings of this study as such support guidelines recommendations for BB as first line agents; within CCBs, verapamil has more data for efficacy than diltiazem. Disopyramide and CMIs appear to be effective add-on agents to BBs, with disopyramide appearing to have greater effect.

This meta-analysis has several limitations, and overall observations need to be considered cautiously due to the observational nature of the studies evaluating negative inotrope therapies (BBs, CCBs, and disopyramide), with these studies being older, with small sample sizes and lacking randomization, blindness, and a control group. In contrast, CMIs which were studied in the contemporary era had randomized controlled trials to support their efficacy. Indirect treatment comparison methods (such as matching-adjusted indirect comparison or network analysis) were not possible because of inability to access individual participate data with the older studies. Additionally, multiple studies evaluating disopyramide and CMIs included background/concurrent treatments with other agents (e.g. BBs or CCBs) that may have contributed to the observed reduction in LVOT gradient. However, for the placebo-controlled trials, we assessed the relative reduction in LVOT gradient induced by the relevant intervention (i.e. compared with placebo) in our analysis to adjust for the background treatments. For the single-arm studies, the baseline LVOT gradient represented a residual gradient after treatment with the first-line medications. Therefore, subsequent changes from these baseline gradients can be mainly attributed to the addition of the new drug that is being evaluated. Of note, this effectively reflects real-world clinical practice, as outlined in the most recent guidelines.

There was significant heterogeneity among the included studies including differing drug doses and duration, follow-up times between baseline and follow-up measurements of LVOT gradient and the methods used to measure the LVOT gradient (invasive vs. Doppler by echocardiogram, with considerable technical differences due to the progress in the last decades). However, published data suggests a close correlation between invasive and echocardiographic measurements of LVOT gradient.^5^ In addition, because of the small number of studies that investigated each individual drug, further sub-group analyses (e.g. based on drug doses) to assess the mentioned heterogeneity were not feasible. The results for some agents, such as diltiazem and some BBs, were constrained by the limited number of studies available. Finally, the influence of different HCM genotypes was not assessed in most of the included studies, except in three mavacamten trials. However, the response to treatment across these trials was similar between individuals with known genetic variants and those without identified mutations.

## Conclusions

Most currently used pharmacological therapies reduced LVOT gradient in oHCM patients, with disopyramide and CMIs showing the highest effect, followed by BBs and CCBs. Different agents within each class had differing efficacy on LVOT gradient reduction. These findings may be used to guide medical management for patients with oHCM.

## Supplementary Material

pvaf036_Supplementary_Data

## Data Availability

The data underlying this article will be shared on reasonable request to the corresponding author.
