# Supplementary material for: Efficacy of pharmacological therapies in reducing outflow tract obstruction in patients with obstructive hypertrophic cardiomyopathy: a systematic review and meta-analysis
Source: Eur Heart J Cardiovasc Pharmacother. 2025 May 16;11(5):469–82. doi: 10.1093/ehjcvp/pvaf036 (PMC12343041; doi:10.1093/ehjcvp/pvaf036)
Supplement: pvaf036_Supplementary_Data [file pvaf036_supplementary_data.docx]

**Supplementary Table 1**. Preferred Reporting Items for Systematic Reviews and Meta-Analyses (PRISMA) statement checklist.

| **Section and Topic** | **Item #** | **Checklist item** | **Location where item is reported** |
| --- | --- | --- | --- |
| **TITLE** | | |  |
| Title | 1 | Identify the report as a systematic review. | 1 |
| **ABSTRACT** | | |  |
| Abstract | 2 | See the PRISMA 2020 for Abstracts checklist. | 4 |
| **INTRODUCTION** | | |  |
| Rationale | 3 | Describe the rationale for the review in the context of existing knowledge. | 3 |
| Objectives | 4 | Provide an explicit statement of the objective(s) or question(s) the review addresses. | 3 |
| **METHODS** | | |  |
| Eligibility criteria | 5 | Specify the inclusion and exclusion criteria for the review and how studies were grouped for the syntheses. | 4, 5 |
| Information sources | 6 | Specify all databases, registers, websites, organisations, reference lists and other sources searched or consulted to identify studies. Specify the date when each source was last searched or consulted. | 4 |
| Search strategy | 7 | Present the full search strategies for all databases, registers and websites, including any filters and limits used. | 4 |
| Selection process | 8 | Specify the methods used to decide whether a study met the inclusion criteria of the review, including how many reviewers screened each record and each report retrieved, whether they worked independently, and if applicable, details of automation tools used in the process. | 4, 5 |
| Data collection process | 9 | Specify the methods used to collect data from reports, including how many reviewers collected data from each report, whether they worked independently, any processes for obtaining or confirming data from study investigators, and if applicable, details of automation tools used in the process. | 5, 6 |
| Data items | 10a | List and define all outcomes for which data were sought. Specify whether all results that were compatible with each outcome domain in each study were sought (e.g. for all measures, time points, analyses), and if not, the methods used to decide which results to collect. | 5, 6 |
|  | 10b | List and define all other variables for which data were sought (e.g. participant and intervention characteristics, funding sources). Describe any assumptions made about any missing or unclear information. | 6 |
| Study risk of bias assessment | 11 | Specify the methods used to assess risk of bias in the included studies, including details of the tool(s) used, how many reviewers assessed each study and whether they worked independently, and if applicable, details of automation tools used in the process. | 6 |
| Effect measures | 12 | Specify for each outcome the effect measure(s) (e.g. risk ratio, mean difference) used in the synthesis or presentation of results. | 6, 7 |
| Synthesis methods | 13a | Describe the processes used to decide which studies were eligible for each synthesis (e.g. tabulating the study intervention characteristics and comparing against the planned groups for each synthesis (item #5)). | 6, 7 |
|  | 13b | Describe any methods required to prepare the data for presentation or synthesis, such as handling of missing summary statistics, or data conversions. | 6, 7 |
|  | 13c | Describe any methods used to tabulate or visually display results of individual studies and syntheses. | 6, 7 |
|  | 13d | Describe any methods used to synthesize results and provide a rationale for the choice(s). If meta-analysis was performed, describe the model(s), method(s) to identify the presence and extent of statistical heterogeneity, and software package(s) used. | 7 |
|  | 13e | Describe any methods used to explore possible causes of heterogeneity among study results (e.g. subgroup analysis, meta-regression). | 7 |
|  | 13f | Describe any sensitivity analyses conducted to assess robustness of the synthesized results. | 7 |
| Reporting bias assessment | 14 | Describe any methods used to assess risk of bias due to missing results in a synthesis (arising from reporting biases). | 6 |
| Certainty assessment | 15 | Describe any methods used to assess certainty (or confidence) in the body of evidence for an outcome. | 6 |
| **RESULTS** | | |  |
| Study selection | 16a | Describe the results of the search and selection process, from the number of records identified in the search to the number of studies included in the review, ideally using a flow diagram. | 8 |
|  | 16b | Cite studies that might appear to meet the inclusion criteria, but which were excluded, and explain why they were excluded. | 8 |
| Study characteristics | 17 | Cite each included study and present its characteristics. | 8, 9 |
| Risk of bias in studies | 18 | Present assessments of risk of bias for each included study. | 8 |
| Results of individual studies | 19 | For all outcomes, present, for each study: (a) summary statistics for each group (where appropriate) and (b) an effect estimate and its precision (e.g. confidence/credible interval), ideally using structured tables or plots. | 8, 9, 10 |
| Results of syntheses | 20a | For each synthesis, briefly summarise the characteristics and risk of bias among contributing studies. | 8, 9, 10 |
|  | 20b | Present results of all statistical syntheses conducted. If meta-analysis was done, present for each the summary estimate and its precision (e.g. confidence/credible interval) and measures of statistical heterogeneity. If comparing groups, describe the direction of the effect. | 8, 9, 10 |
|  | 20c | Present results of all investigations of possible causes of heterogeneity among study results. | 8, 9, 10 |
|  | 20d | Present results of all sensitivity analyses conducted to assess the robustness of the synthesized results. | 8, 9, 10 |
| Reporting biases | 21 | Present assessments of risk of bias due to missing results (arising from reporting biases) for each synthesis assessed. | 8 |
| Certainty of evidence | 22 | Present assessments of certainty (or confidence) in the body of evidence for each outcome assessed. | 8 |
| **DISCUSSION** | | |  |
| Discussion | 23a | Provide a general interpretation of the results in the context of other evidence. | 10, 11 |
|  | 23b | Discuss any limitations of the evidence included in the review. | 13, 14, 15 |
|  | 23c | Discuss any limitations of the review processes used. | 14, 15 |
|  | 23d | Discuss implications of the results for practice, policy, and future research. | 13, 14 |
| **OTHER INFORMATION** | | |  |
| Registration and protocol | 24a | Provide registration information for the review, including register name and registration number, or state that the review was not registered. | 4 |
|  | 24b | Indicate where the review protocol can be accessed, or state that a protocol was not prepared. | 4 |
|  | 24c | Describe and explain any amendments to information provided at registration or in the protocol. | 4 |
| Support | 25 | Describe sources of financial or non-financial support for the review, and the role of the funders or sponsors in the review. | 16 |
| Competing interests | 26 | Declare any competing interests of review authors. | 16 |
| Availability of data, code and other materials | 27 | Report which of the following are publicly available and where they can be found: template data collection forms; data extracted from included studies; data used for all analyses; analytic code; any other materials used in the review. | 16 |

**Supplementary Table 2.** Full literature search strategy used to identify the relevant included studies.

| **PubMed** | ((((((((("Adrenergic beta-Antagonists"[Mesh]) OR ("Calcium Channel Blockers"[Mesh])) OR ("Disopyramide"[Mesh])) OR ("Cardiac Myosins"[Mesh])) OR (Beta blocker*)) OR (Myosin inhibitor*)) OR (Negative inotropic)) OR (Pindolol)) OR (((((((((((Metoprolol) OR (Propranolol)) OR (Bisoprolol)) OR (Atenolol)) OR (Carvedilol)) OR (Nebivolol)) OR (Verapamil)) OR (Diltiazem)) OR (Nifedipine)) OR (Nisoldipine)) OR (Nadolol))) AND ((((("Cardiomyopathy, Hypertrophic, Familial"[Mesh]) OR ("Cardiomyopathy, Hypertrophic"[Mesh])) OR (hypertrophic cardiomyopathy)) OR (HCM)) OR (Subaortic Stenosis)) | 2,263 results |
| --- | --- | --- |
| **Scopus** | ( ( TITLE-ABS-KEY ( calcium AND channel AND blockers ) OR TITLE-ABS-KEY ( adrenergic AND beta-antagonists ) OR TITLE-ABS-KEY ( disopyramide ) OR TITLE-ABS-KEY ( cardiac AND myosins ) OR TITLE-ABS-KEY ( beta AND blocker* ) OR TITLE-ABS-KEY ( myosin AND inhibitor* ) OR TITLE-ABS-KEY ( negative AND inotropic ) OR TITLE-ABS-KEY ( pindolol ) OR TITLE-ABS-KEY ( metoprolol ) OR TITLE-ABS-KEY ( propranolol ) OR TITLE-ABS-KEY ( bisoprolol ) OR TITLE-ABS-KEY ( atenolol ) OR TITLE-ABS-KEY ( carvedilol ) OR TITLE-ABS-KEY ( nebivolol ) OR TITLE-ABS-KEY ( verapamil ) OR TITLE-ABS-KEY ( diltiazem ) OR TITLE-ABS-KEY ( nifedipine ) OR TITLE-ABS-KEY ( nisoldipine ) OR TITLE-ABS-KEY ( nadolol ) ) ) AND ( ( TITLE-ABS-KEY ( hypertrophic AND cardiomyopathy ) OR TITLE-ABS-KEY ( hcm ) OR TITLE-ABS-KEY ( subaortic AND stenosis ) ) ) | 5,902 results |

**Supplementary Table 3**: List of the included studies.

| **Study ID** | **Year of publication** |
| --- | --- |
| Monda *et al.* (1) | 2022 |
| Dybro *et al. (2)* | 2021 |
| Haruki *et al. (3)* | 2015 |
| Sherrid *et al. (4)* | 2013 |
| Nistri *et al. (5)* | 2012 |
| Kajimoto *et al. (6)* | 2010 |
| Sherrid *et al. (7)* | 2005 |
| Betocchi *et al. (8)* | 1996 |
| Matsubara *et al. (9)* | 1995 |
| Kimball *et al. (10)* | 1993 |
| Dimitrow *et al. (11)* | 1993 |
| Millaire *et al. (12)* | 1992 |
| Pollick *et al. (13)* | 1988 |
| Pollick *et al. (14)* | 1988 |
| Sherrid *et al. (15)* | 1988 |
| Anderson *et al. (16)* | 1984 |
| Bonow *et al. (17)* | 1983 |
| Tendera *et al. (18)* | 1983 |
| Landmark *et al. (19)* | 1982 |
| Pollick *et al. (20)* | 1982 |
| Storstein *et al. (21)* | 1981 |
| Kaltenbach *et al. (22)* | 1979 |
| Rosing *et al. (23)* | 1979 |
| Todde *et al. (24)* | 2024 |
| Abood *et al. (25)* | 2024 |
| Desai *et al. (26)* | 2024 |
| Ramonfaur *et al. (27)* | 2024 |
| Reza *et al. (28)* | 2024 |
| Roehl *et al. (29)* | 2024 |
| Wessly *et al. (30)* | 2023 |
| EXPLORER-CN (31) | 2023 |
| EXPLORER-HCM (32) | 2020 |
| PIONEER-HCM (33) | 2019 |
| VALOR-HCM (34) | 2022 |
| SEQUOIA-HCM (35) | 2024 |
| REDWOOD-HCM (36) | 2023 |
| REDWOOD-HCM (cohort 3) (37) | 2023 |

**Supplementary Table 4**: summary of the frequency of adverse events reported in the included studies.

| **Study ID** | **Side Effects** |
| --- | --- |
| Monda et al, 2022 | (1) intolerance |
| Dybro et al, 2021 | (3) dizziness, (1) fatigue, (2) cold hands/feet, (2) diarrhea, (1) tiredness, and (1) tingling hands/feet |
| Haruki et al, 2015 | None |
| Sherrid et al, 2013 | (1) torsade de pointes; (1) heart block, (1) urinary hesitancy, and (1) urinary retention |
| Nistri et al, 2012 | NA |
| Kajimoto et al, 2010 (A) | NA |
| Kajimoto et al, 2010 (B) |  |
| Kajimoto et al, 2010 (C) |  |
| Sherrid et al, 2005 (A) | (5) dry mouth, and (3) symptoms related to prostatism |
| Sherrid et al, 2005 (B) |  |
| Betocchi et al, 1996 | NA |
| Matsubara et al, 1995 | None |
| Kimball et al, 1993 | NA |
| Dimitrow et al, 1993 | NA |
| Millaire et al, 1992 | NA |
| Pollick et al, 1988 (A) | (8) dry mouth and blurred vision |
| Pollick et al, 1988 (B) | none |
| Pollick et al, 1988 (C) | (1) felt presyncopal |
| Sherrid et al, 1988 | (1) Mobitz II atrioventricular block, and (1) mild cholinergic side effects |
| Anderson et al, 1984 | None |
| Bonow et al, 1983 | NA |
| Tendera et al, 1983 | NA |
| Landmark et al, 1982 (B) | NA |
| Pollick et al, 1982 | Dry mouth and mild blurred vision |
| Storstein et al, 1981 | NA |
| Kaltenbach et al, 1979 | (1) Atrioventricular block, and (5) dizziness, nausea, headache, or chest pain |
| Rosing et al, 1979 | (2) hypotension, (27) prolonged PR, (2) occasional non-conducted sinus beats |
| Todde et al, 2024 (A) | (2) anticholinergic symptoms, and (4) QTc prolongation |
| Todde et al, 2024 (B) |  |
| Abood et al, 2024 | (2) fatigue, and no major side effects |
| Desai et al, 2024 | (3) ejection fraction reduction, (1) hepatic decompensation, (1) stroke not due to atrial fibrillation, (1) pulmonary embolism |
| Ramonfaur et al, 2024 | (3) ejection fraction reduction, (1) dizziness, (1) asthma exacerbation, (1) new onset atrial fibrillation, (3) recurrent atrial fibrillation |
| Reza et al, 2024 | (4) ejection fraction reduction, (1) severe mitral regurgitation, and (1) atrial fibrillation with heart failure |
| Roehl et al, 2024 | NA |
| Wessly et al, 2023 | (2) atrial fibrillation |
| EXPLORER-CN, 2023 | Total treatment emergent AEs in 45 in mavacamten versus 24 in placebo |
| EXPLORER-HCM, 2020 | Treatment emergent AEs in 108 in mavacamten versus vs 101 in placebo |
| PIONEER-HCM, 2019 (A) | (1) ventricular tachycardia, (3) atrial fibrillation, (3) headache, (3) ejection fraction drop, (2) rash, (2) URTI, (2) UTI, (2) exertional dyspnea, (2) fatigue, (2) nausea, and (1) dizziness |
| PIONEER-HCM, 2019 (B) | (4) ventricular tachycardia, (1) atrial fibrillation, (2) headache, (2) angina, (2) URTI, (2) fatigue, and (3) dizziness |
| VALOR-HCM, 2022 | At least one on-treatment AE in 41 in mavacamten versus 34 in placebo |
| SEQUOIA-HCM, 2024 | Any AEs in aficamten 105 versus 99 in placebo |
| REDWOOD-HCM, 2023 | Total treatment emergent AEs in 26 in aficamten versus 40 in placebo. |
| REDWOOD-HCM (cohort 3), 2023 | None |

Abbreviations: NA: not available, AE: adverse event, URTI: upper respiratory tract infection, UTI: urinary tract infection.

**Supplementary Table 5.** Study risk of bias assessment using ROBINS-I tool for the included observational/non-randomized trials and RoB 2 tool for included randomized clinical trials.

| **ROBINS-I tool** | | | | | | | | |
| --- | --- | --- | --- | --- | --- | --- | --- | --- |
| **Author, year** | **Bias due to confounding** | **Bias in selection of participants into the study** | **Bias in classification of interventions** | **Bias due to deviations from intended interventions** | **Bias due to missing data** | **Bias in measurement of outcomes** | **Bias in selection of the reported result** | **Overall bias** |
| Monda et al, 2022 | Moderate | Low | Low | Low | Low | Moderate | Low | Moderate |
| Haruki et al, 2015 | Serious | Moderate | Low | Low | Low | Moderate | Low | Serious |
| Sherrid et al, 2013 | Moderate | Low | Low | Low | Low | Moderate | Low | Moderate |
| Nistri et al, 2012 | Moderate | Low | Low | Low | Low | Moderate | Low | Moderate |
| Kajimoto et al, 2010 | Serious | Low | Low | Low | Low | Low | Low | Serious |
| Sherrid et al, 2005 | Moderate | Low | low | Moderate | low | Moderate | Moderate | Moderate |
| Betocchi et al, 1996 | Serious | Low | Low | Low | Low | Moderate | Low | Serious |
| Matsubara et al, 1995 | Serious | Low | Low | Low | Low | Moderate | Moderate | Serious |
| Kimball et al, 1993 | Serious | Low | Low | Low | Low | Moderate | Low | Serious |
| Dimitrow et al, 1993 | Serious | Low | Low | Low | Low | Moderate | Low | Serious |
| Millaire et al, 1992 | Serious | Low | Low | Low | Moderate | Moderate | Low | Serious |
| Pollick et al, 1988 (A) | Serious | Low | Low | Low | Low | Low | Low | Serious |
| Sherrid et al, 1988 | Serious | Low | Low | Low | Low | Moderate | Moderate | Serious |
| Anderson et al, 1984 | Serious | Low | Low | low | Moderate | Moderate | Moderate | Serious |
| Bonow et al, 1983 | Serious | Low | Low | Low | Low | Moderate | Moderate | Serious |
| Tendera et al, 1983 | Serious | Low | Low | Low | Low | Low | Low | Serious |
| Landmark et al, 1982 | Serious | Low | Low | Low | Low | Low | Low | Serious |
| Pollick et al, 1982 | Serious | Low | Low | Low | Low | Low | Moderate | Serious |
| Storstein et al, 1981 | Serious | Low | Low | Low | Low | Low | Low | Serious |
| Kaltenbach et al, 1979 | Serious | Low | Low | Low | Moderate | Moderate | Moderate | Serious |
| Rosing et al, 1979 | Serious | Low | Low | Low | Moderate | Low | Low | Serious |
| Todde et al, 2024 | Moderate | Low | Low | Low | Low | Moderate | Moderate | Moderate |
| Abood et al, 2024 | Serious | Low | Low | Low | Low | Moderate | Low | Serious |
| Desai et al, 2024 | Serious | Low | Low | Low | Low | Moderate | Low | Serious |
| Ramonfaur et al, 2024 | Moderate | Low | Low | Low | Low | Moderate | Low | Moderate |
| Reza et al, 2024 | Serious | Low | Low | Low | Low | Moderate | Low | Serious |
| Roehl et al, 2024 | Serious | Moderate | Low | Low | Low | Low | Low | Serious |
| Wessly et al, 2023 | Serious | Low | Low | Low | Low | Moderate | Low | Serious |
| REDWOOD-HCM (cohort 3) | Moderate | Low | Low | Low | Low | Moderate | Low | Moderate |
| PIONEER-HCM | Moderate | Low | Low | Low | Low | Moderate | Low | Moderate |
| **RoB2 tool** | | | | | | | | |
| **Author, year** | **Randomization process** | **Effect of assignment to intervention** | **Effect of adhering to intervention** | **Missing outcome data** | **Measurement of the outcome** | **Selection of the reported result** | **Overall risk of bias** | |
| Dybro et al, 2021 | Low | Low | Low | Low | Low | Low | Low | |
| Pollick et al, 1988 (B) | Low | Low | Low | Low | Some concerns | Low | Some concerns | |
| EXPLORER-CN | Low | Low | Low | Low | Low | Low | Low | |
| EXPLORER-HCM | Low | Low | Low | Low | Low | Low | Low | |
| VALOR-HCM | Low | Low | Low | Low | Low | Low | Low | |
| SEQUOIA-HCM | Low | Low | Low | Low | Low | Low | Low | |
| REDWOOD-HCM | Low | Low | Low | Low | Low | Low | Low | |


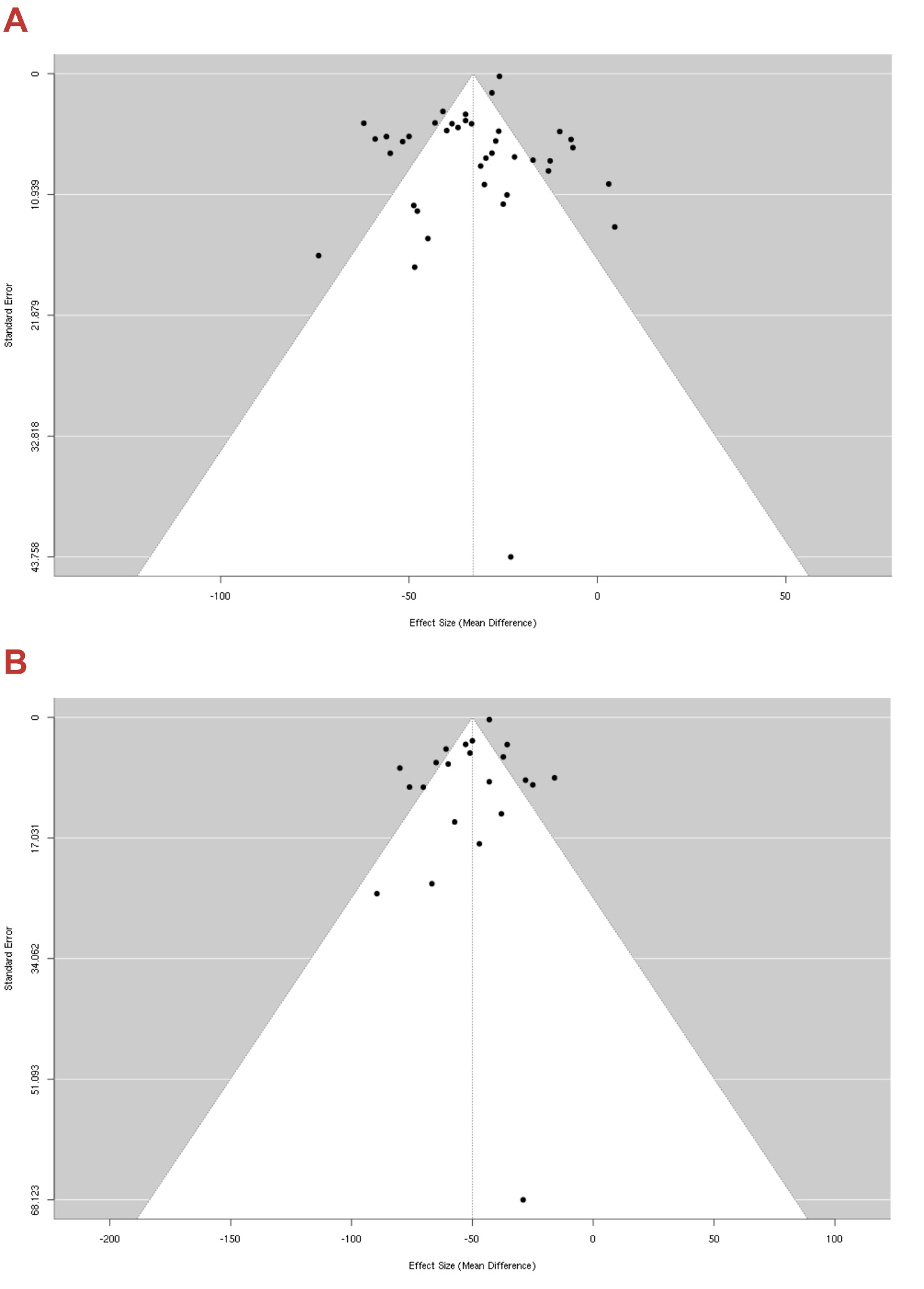


**Supplementary Figure 1**. Funnel plot showing publication bias in the included studies that investigated the effect of pharmacological therapies on reduction of: (A) resting and (B) provoked LVOT gradients in oHCM.

**REFERENCES**:

1. Monda E, Lioncino M, Palmiero G, Franco F, Rubino M, Cirillo A, et al. Bisoprolol for treatment of symptomatic patients with obstructive hypertrophic cardiomyopathy. The BASIC (bisoprolol AS therapy in hypertrophic cardiomyopathy) study. *Int J Cardiol*. 2022 May 1;**354**:22-8. PubMed PMID: 35278578. Epub 20220310.

2. Dybro AM, Rasmussen TB, Nielsen RR, Andersen MJ, Jensen MK, Poulsen SH. Randomized Trial of Metoprolol in Patients With Obstructive Hypertrophic Cardiomyopathy. *J Am Coll Cardiol*. 2021 Dec 21;**78**(25):2505-17. PubMed PMID: 34915981.

3. Haruki S, Minami Y, Suzuki A, Hagiwara N. Effects of flecainide on left ventricular pressure gradient and symptoms in obstructive hypertrophic cardiomyopathy: a comparison of flecainide and disopyramide. *Heart Vessels*. 2015 Sep;**30**(5):604-10. PubMed PMID: 24917414. Epub 20140612.

4. Sherrid MV, Shetty A, Winson G, Kim B, Musat D, Alviar CL, et al. Treatment of obstructive hypertrophic cardiomyopathy symptoms and gradient resistant to first-line therapy with beta-blockade or verapamil. *Circ Heart Fail*. 2013 Jul;**6**(4):694-702. PubMed PMID: 23704138. Epub 20130523.

5. Nistri S, Olivotto I, Maron MS, Ferrantini C, Coppini R, Grifoni C, et al. beta Blockers for prevention of exercise-induced left ventricular outflow tract obstruction in patients with hypertrophic cardiomyopathy. *Am J Cardiol*. 2012 Sep 1;**110**(5):715-9. PubMed PMID: 22633205. Epub 20120524.

6. Kajimoto K, Imai T, Minami Y, Kasanuki H. Comparison of acute reduction in left ventricular outflow tract pressure gradient in obstructive hypertrophic cardiomyopathy by disopyramide versus pilsicainide versus cibenzoline. *Am J Cardiol*. 2010 Nov 1;**106**(9):1307-12. PubMed PMID: 21029829.

7. Sherrid MV, Barac I, McKenna WJ, Elliott PM, Dickie S, Chojnowska L, et al. Multicenter study of the efficacy and safety of disopyramide in obstructive hypertrophic cardiomyopathy. *J Am Coll Cardiol*. 2005 Apr 19;**45**(8):1251-8. PubMed PMID: 15837258.

8. Betocchi S, Piscione F, Losi MA, Pace L, Boccalatte M, Perrone-Filardi P, et al. Effects of diltiazem on left ventricular systolic and diastolic function in hypertrophic cardiomyopathy. *Am J Cardiol*. 1996 Aug 15;**78**(4):451-7. PubMed PMID: 8752192.

9. Matsubara H, Nakatani S, Nagata S, Ishikura F, Katagiri Y, Ohe T, et al. Salutary effect of disopyramide on left ventricular diastolic function in hypertrophic obstructive cardiomyopathy. *J Am Coll Cardiol*. 1995 Sep;**26**(3):768-75. PubMed PMID: 7642872.

10. Kimball BP, Bui S, Wigle ED. Acute dose-response effects of intravenous disopyramide in hypertrophic obstructive cardiomyopathy. *Am Heart J*. 1993 Jun;**125**(6):1691-7. PubMed PMID: 8498312.

11. Dimitrow PP, Dubiel JS. Effects on left ventricular function of pindolol added to verapamil in hypertrophic cardiomyopathy. *Am J Cardiol*. 1993 Feb 1;**71**(4):313-6. PubMed PMID: 8427174.

12. Millaire A, Goullard L, Decoulx E, de Groote P, Houdas Y, Ducloux G. Efficiency of disopyramide in hypertrophic cardiomyopathy during stress states. *Am J Cardiol*. 1992 Feb 1;**69**(4):423-4. PubMed PMID: 1734662.

13. Pollick C, Kimball B, Henderson M, Wigle ED. Disopyramide in hypertrophic cardiomyopathy. I. Hemodynamic assessment after intravenous administration. *Am J Cardiol*. 1988 Dec 1;**62**(17):1248-51. PubMed PMID: 3195486.

14. Pollick C. Disopyramide in hypertrophic cardiomyopathy. II. Noninvasive assessment after oral administration. *Am J Cardiol*. 1988 Dec 1;**62**(17):1252-5. PubMed PMID: 3057852.

15. Sherrid M, Delia E, Dwyer E. Oral disopyramide therapy for obstructive hypertrophic cardiomyopathy. *Am J Cardiol*. 1988 Nov 15;**62**(16):1085-8. PubMed PMID: 3189171.

16. Anderson DM, Raff GL, Ports TA, Brundage BH, Parmley WW, Chatterjee K. Hypertrophic obstructive cardiomyopathy. Effects of acute and chronic verapamil treatment on left ventricular systolic and diastolic function. *Br Heart J*. 1984 May;**51**(5):523-9. PubMed PMID: 6539120. Pubmed Central PMCID: PMC481543.

17. Bonow RO, Ostrow HG, Rosing DR, Cannon RO, 3rd, Lipson LC, Maron BJ, et al. Effects of verapamil on left ventricular systolic and diastolic function in patients with hypertrophic cardiomyopathy: pressure-volume analysis with a nonimaging scintillation probe. *Circulation*. 1983 Nov;**68**(5):1062-73. PubMed PMID: 6684510.

18. Tendera M, Polonski L, Kozielska E. Left ventricular end-diastolic pressure-volume relationships in hypertrophic cardiomyopathy. Changes induced by verapamil. *Chest*. 1983 Jul;**84**(1):54-7. PubMed PMID: 6683147.

19. Landmark K, Sire S, Thaulow E, Amlie JP, Nitter-Hauge S. Haemodynamic effects of nifedipine and propranolol in patients with hypertrophic obstructive cardiomyopathy. *Br Heart J*. 1982 Jul;**48**(1):19-26. PubMed PMID: 7200796. Pubmed Central PMCID: PMC481196.

20. Pollick C. Muscular subaortic stenosis: hemodynamic and clinical improvement after disopyramide. *N Engl J Med*. 1982 Oct 14;**307**(16):997-9. PubMed PMID: 7202121.

21. Storstein L, Abrahamsen AM, Storstein O. Hemodynamic effects of strophanthin and pindolol in patients with hypertrophic cardiomyopathy. *Eur Heart J*. 1981 Aug;**2**(4):297-305. PubMed PMID: 6117467.

22. Kaltenbach M, Hopf R, Kober G, Bussmann WD, Keller M, Petersen Y. Treatment of hypertrophic obstructive cardiomyopathy with verapamil. *Br Heart J*. 1979 Jul;**42**(1):35-42. PubMed PMID: 573129. Pubmed Central PMCID: PMC482109.

23. Rosing DR, Kent KM, Borer JS, Seides SF, Maron BJ, Epstein SE. Verapamil therapy: a new approach to the pharmacologic treatment of hypertrophic cardiomyopathy. I. Hemodynamic effects. *Circulation*. 1979 Dec;**60**(6):1201-7. PubMed PMID: 574066.

24. Todde G, Dei LL, Polizzi R, Gabrielli D, Canciello G, Romano S, et al. Long-term follow-up study on obstructive hypertrophic cardiomyopathy patients treated with disopyramide: evidences of a notable trend in symptom control within a real-world clinical setting. *Front Cardiovasc Med*. 2024;**11**:1416600. PubMed PMID: 39175632. Pubmed Central PMCID: PMC11340511. Epub 20240814.

25. Abood Z, Jan MF, Ashraf M, Kroboth S, Sanders H, Schweitzer M, et al. Mavacamten in real-life practice: Initial experience at a hypertrophic cardiomyopathy centre. *ESC Heart Fail*. 2025 Feb;**12**(1):672-6. PubMed PMID: 39137157. Pubmed Central PMCID: PMC11769649. Epub 20240813.

26. Desai MY, Hajj-Ali A, Rutkowski K, Ospina S, Gaballa A, Emery M, et al. Real-world experience with mavacamten in obstructive hypertrophic cardiomyopathy: Observations from a tertiary care center. *Prog Cardiovasc Dis*. 2024 Sep-Oct;**86**:62-8. PubMed PMID: 38354765. Epub 20240213.

27. Ramonfaur D, Gasperetti A, Blake VE, Rivers B, Kassamali AA, Kasper EK, et al. Eighteen-Month Real-World Experience Using Mavacamten for Treatment of Obstructive Hypertrophic Cardiomyopathy in a Racially Diverse Population. *J Am Heart Assoc*. 2024 Aug 6;**13**(15):e034069. PubMed PMID: 39082420. Pubmed Central PMCID: PMC11964038. Epub 20240731.

28. Reza N, Dubey A, Carattini T, Marzolf A, Hornsby N, de Feria A, et al. Real-World Experience and 36-Week Outcomes of Patients With Symptomatic Obstructive Hypertrophic Cardiomyopathy Treated With Mavacamten. *JACC Heart Fail*. 2024 Jun;**12**(6):1123-5. PubMed PMID: 38661589. Pubmed Central PMCID: PMC11156526. Epub 20240407.

29. Roehl KM, Farina JM, Suppah M, Arsanjani R, Lester SJ, Geske JB, et al. Predictors of short-term and long-term effects of mavacamten in obstructive hypertrophic cardiomyopathy. *Prog Cardiovasc Dis*. 2024 Sep-Oct;**86**:86-8. PubMed PMID: 38782245. Epub 20240521.

30. Wessly P, Lazzara GE, Buergler JM, Nagueh SF. Early Observations on Effects of Mavacamten on Left Atrial Function in Obstructive Hypertrophic Cardiomyopathy Patients. *JACC Cardiovasc Imaging*. 2023 Dec;**16**(12):1633-4. PubMed PMID: 37452821. Epub 20230712.

31. Tian Z, Li L, Li X, Wang J, Zhang Q, Li Z, et al. Effect of Mavacamten on Chinese Patients With Symptomatic Obstructive Hypertrophic Cardiomyopathy: The EXPLORER-CN Randomized Clinical Trial. *JAMA Cardiol*. 2023 Oct 1;**8**(10):957-65. PubMed PMID: 37639259. Pubmed Central PMCID: PMC10463173.

32. Olivotto I, Oreziak A, Barriales-Villa R, Abraham TP, Masri A, Garcia-Pavia P, et al. Mavacamten for treatment of symptomatic obstructive hypertrophic cardiomyopathy (EXPLORER-HCM): a randomised, double-blind, placebo-controlled, phase 3 trial. *Lancet*. 2020 Sep 12;**396**(10253):759-69. PubMed PMID: 32871100. Epub 20200829.

33. Heitner SB, Jacoby D, Lester SJ, Owens A, Wang A, Zhang D, et al. Mavacamten Treatment for Obstructive Hypertrophic Cardiomyopathy: A Clinical Trial. *Ann Intern Med*. 2019 Jun 4;**170**(11):741-8. PubMed PMID: 31035291. Epub 20190430.

34. Desai MY, Owens A, Geske JB, Wolski K, Naidu SS, Smedira NG, et al. Myosin Inhibition in Patients With Obstructive Hypertrophic Cardiomyopathy Referred for Septal Reduction Therapy. *J Am Coll Cardiol*. 2022 Jul 12;**80**(2):95-108. PubMed PMID: 35798455.

35. Maron MS, Masri A, Nassif ME, Barriales-Villa R, Arad M, Cardim N, et al. Aficamten for Symptomatic Obstructive Hypertrophic Cardiomyopathy. *N Engl J Med*. 2024 May 30;**390**(20):1849-61. PubMed PMID: 38739079. Epub 20240513.

36. Maron MS, Masri A, Choudhury L, Olivotto I, Saberi S, Wang A, et al. Phase 2 Study of Aficamten in Patients With Obstructive Hypertrophic Cardiomyopathy. *J Am Coll Cardiol*. 2023 Jan 3;**81**(1):34-45. PubMed PMID: 36599608.

37. Owens AT, Masri A, Abraham TP, Choudhury L, Rader F, Symanski JD, et al. Aficamten for Drug-Refractory Severe Obstructive Hypertrophic Cardiomyopathy in Patients Receiving Disopyramide: REDWOOD-HCM Cohort 3. *J Card Fail*. 2023 Nov;**29**(11):1576-82. PubMed PMID: 37473912. Epub 20230718.
